# RETRACTED: Ghose et al. Development and Evaluation of Polymeric Nanosponge Hydrogel for Terbinafine Hydrochloride: Statistical Optimization, In Vitro and In Vivo Studies. *Polymers* 2020, *12*, 2903

**DOI:** 10.3390/polym17233092

**Published:** 2025-11-21

**Authors:** Aditee Ghose, Bushra Nabi, Saleha Rehman, Shadab Md, Nabil A. Alhakamy, Osama A. A. Ahmad, Sanjula Baboota, Javed Ali

**Affiliations:** 1 Department of Pharmaceutics, School of Pharmaceutical Education and Research, Jamia Hamdard, New Delhi 110062, India; aditeeghose@gmail.com (A.G.); nabibushra79@gmail.com (B.N.); saleharehman90@gmail.com (S.R.); sbaboota@jamiahamdard.ac.in (S.B.); 2 Department of Pharmaceutics, Faculty of Pharmacy, King Abdulaziz University, Jeddah 21589, Saudi Arabia; shaque@kau.edu.sa (S.M.); nalhakamy@kau.edu.sa (N.A.A.); oaahmed@kau.edu.sa (O.A.A.A.); 3 Center of Excellence for Drug Research & Pharmaceutical Industries, King Abdulaziz University, Jeddah 21589, Saudi Arabia

The journal retracts the article “Development and Evaluation of Polymeric Nanosponge Hydrogel for Terbinafine Hydrochloride: Statistical Optimization, In Vitro and In Vivo Studies” [1] cited above.

Following publication, concerns were brought to the attention of the Editorial Office regarding the integrity of an image presented in this publication [1].

Adhering to our standard procedure, an investigation was conducted by the Editorial Office and Editorial Board that identified indications of inappropriate image editing contained within Figure 7 in this publication [1]. While the authors collaborated within this process, raw material meeting the journal’s Original Images Requirements could not be provided for Editorial Board evaluation. Consequently, the Editorial Board has lost confidence in the reliability of the findings and has decided to retract this publication, as per MDPI’s retraction policy (MDPI | Research and Publication Ethics).

This retraction was approved by the Editor-in-Chief of the journal *Polymers*.

The authors do not agree with this retraction.
